# Supplementary material for: Gut Microbiota Reshaped by Pectin Treatment Improves Liver Steatosis in Obese Mice
Source: Nutrients. 2021 Oct 22;13(11):3725. doi: 10.3390/nu13113725 (PMC8621973; doi:10.3390/nu13113725)
Supplement: Supplementary file 1 [file nutrients-13-03725-s001.zip › nutrients-1394036-supplementary.pdf]

Supplementary Materials: Figure S1: Effect of pectin in normal diet and high fat diet fed mice, Figure S2: The preventive effect of pectin is dose dependent, Figure S3: IM diversity, Figure S4: FMT from mice fed with HFD and pectin is sufficient to induce browning of WAT in recipient HFD fed mice, Table 1: List of oligonucleotides used in qPCR, Table S2: GC/MS and quantitative parameters of SCFAs analysis.

Figure S1

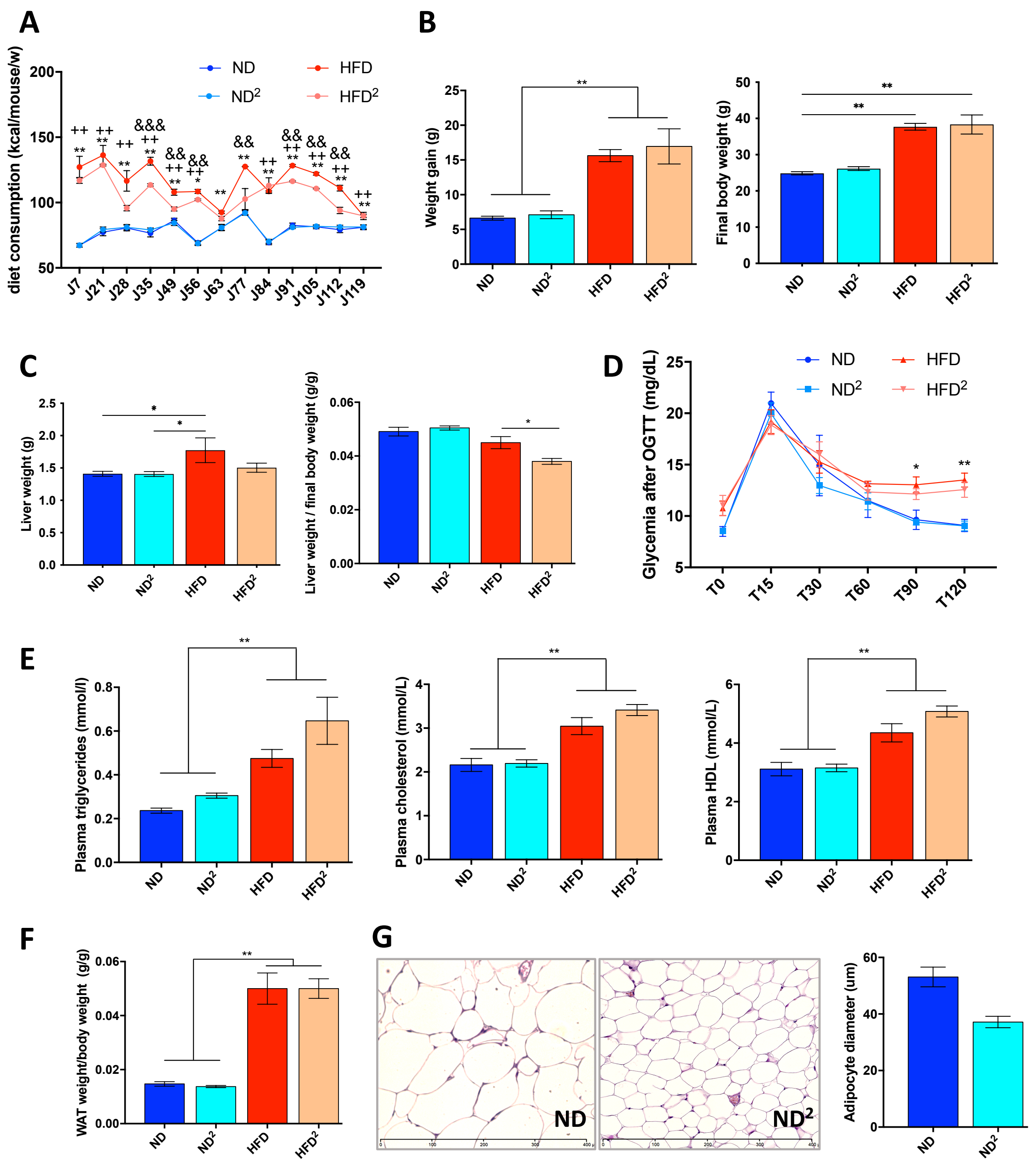

Figure S1. Effect of pectin in normal diet and high fat diet fed mice. Mice were fed with normal diet (ND), ND with pectin 2% (ND^2), high fat diet (HFD) and HFD with pectin 2% (HFD^2) diets for 16 weeks. **(A)** Diet consumption throughout the 16 weeks. **(B)** Total body weight gain after 16 weeks of diet and final body weight. **(C)** Liver weight and Liver weight / body weight ratio. **(D)** Curves of glycemia after an oral glucose tolerance test. **(E)** Post-prandial plasma triglycerides, HDL-cholesterol and total cholesterol. **(F)** White adipose tissue (WAT) weight/body weight ratio. **(G)** WAT sections of ND or ND^2 mice stained with hematoxylin-eosin (scale 400 µm) and histomorphometric analysis of adipocyte diameter. Data represent the mean±SEM of 8 mice, *: $p<0.05$, **: $p<0.01$.

Figure S2

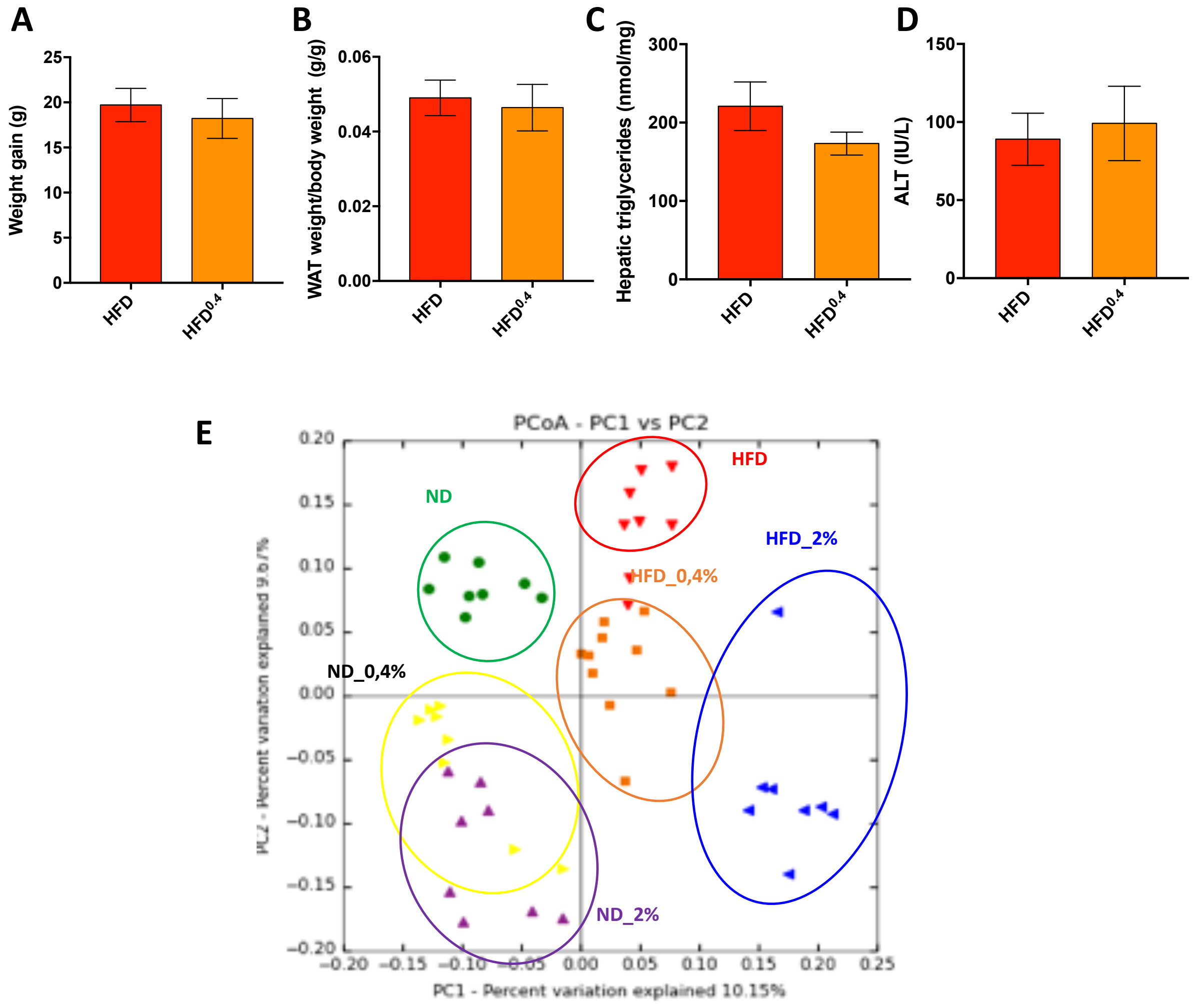

Figure S2. The preventive effect of pectin is dose dependent. Mice were fed with a high-fat diet with or without 0.4% of pectin (HFD, $HFD^{0.4}$) for 16 weeks. **(A)** Total body weight gain at 16 weeks. **(B)** White adipose tissue weight / body weight ratio. **(C)** Hepatic triglycerides content. (**D)** Plasma ALT. **(E)** Unweighted Unifrac distances showing differences of the intestinal microbiota composition even with a low dose of pectin; green=ND, yellow=$ND^{0.4}$, purple=ND^2, red=HFD, orange= $HFD^{0.4}$, blue= HFD^2. Data represent the mean±SEM of 8 mice.

Figure S3

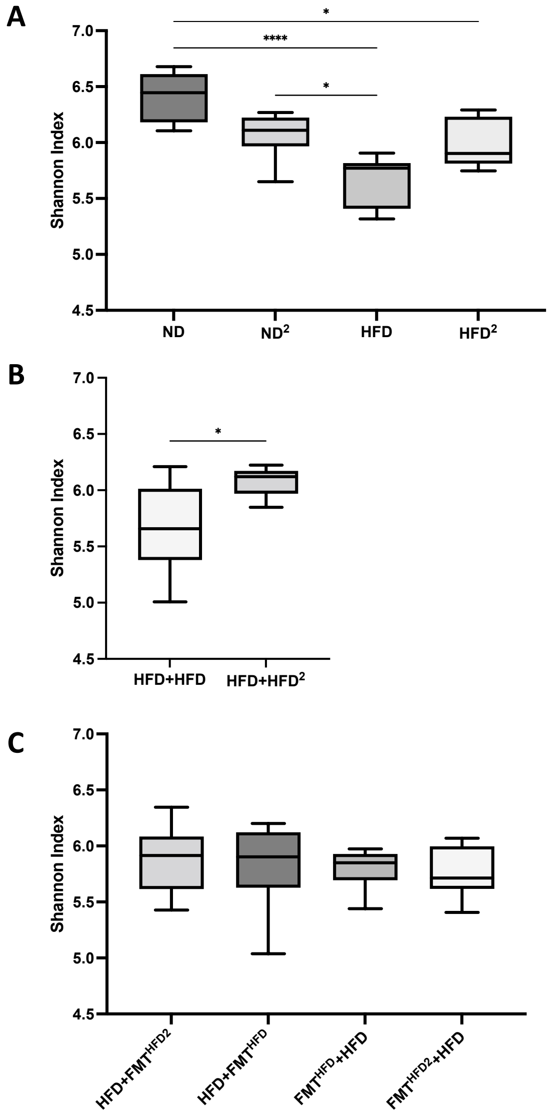

Figure S3. Intestinal microbiota diversity. Box plots showing alpha diversity based on the Shannon Index. **(A)** Mice were fed with normal diet (ND), ND with pectin 2% (ND^2), high fat diet (HFD) and HFD with pectin 2% (HFD^2) diets for 16 weeks.
(B) Mice were fed with HFD for 16 weeks and received pectin supplementation in the HFD (2%) from week 16 to week 24 as curative treatment ($HFD+HFD^2$) compared to mice who did not received pectin (HFD+HFD). **(C)** Mice received a preventive FMT before 16 weeks of HFD from donor mice fed a HFD (FMT^{HFD}+HFD) or from donor mice fed a HFD^2 (FMT^{HFD2}+ HFD). Obese mice received a curative FMT before 8 supplementary weeks of HFD from donor mice fed a HFD (HFD+FMT^{HFD}) or from donor mice fed a HFD^2 (HFD+FMT^{HFD2}). The non parametric Kruskall-Wallis test with Dunn's multiple comparison post-hoc test (A,C) or the Mann-Whitney (B) test were used, $^{*}p < 0.05$.

Figure S4

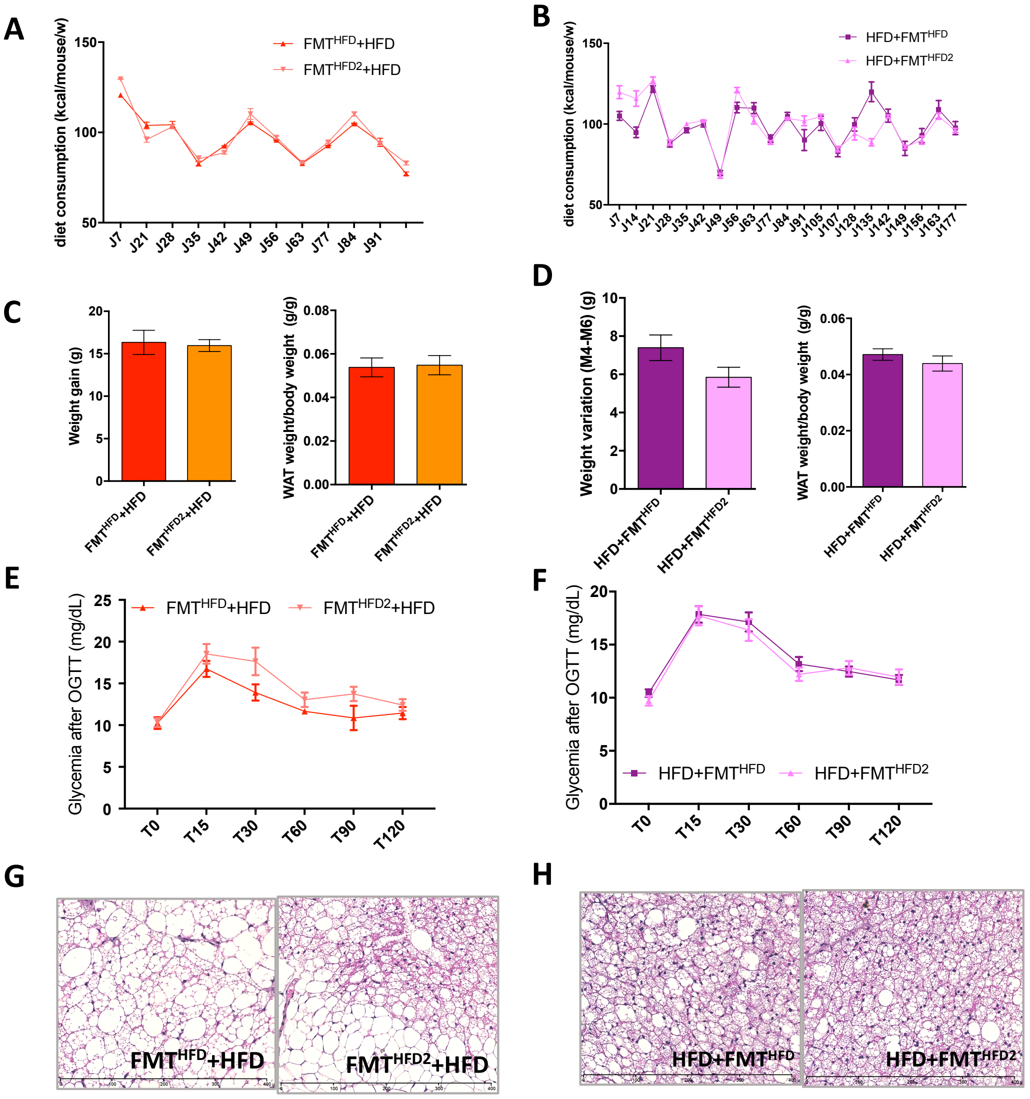

Figure S4. FMT from mice fed with HFD and pectin is sufficient to induce browning of WAT in recipient HFD fed mice. Mice received a preventive FMT along with 16 weeks of HFD from donor mice fed a HFD (FMT^{HFD}+HFD) or from donor mice fed a HFD2 (FMT^{HFD2}+ HFD). Obese mice received a curative FMT during 8 supplementary weeks of HFD from donor mice fed a HFD (HFD+FMT^{HFD}) or from donor mice fed a HFD2 (HFD+FMT^{HFD2}). **(A)** 16 weeks evolution of diet consumption of mice fed with HFD and receiving FMT as preventive treatment. **(B)** 24 weeks evolution of diet consumption of mice fed with HFD and receiving from 16 weeks to 24 weeks FMT as curative treatment. **(C)** Weight gain of FMT^{HFD}+HFD or FMT^{HFD2}+HFD mice (left panel) and white adipose tissue weight/body weight ratio (right panel). **(D)** weight variation between 16 and 24 weeks of HFD+FMT^{HFD} or HFD+FMT^{HFD2} mice (left panel) and white adipose tissue weight/body weight ratio (right panel). **(E)** Curves of glycemia after an oral glucose tolerance test in FMT^{HFD}+HFD or FMT^{HFD2}+HFD mice at 16weeks and **(F)** in HFD+FMT^{HFD} or HFD+FMT^{HFD2} mice at 24 weeks. (G) Brown adipose tissue sections stained with hematoxylin-eosin (scale 400 µm) in FMT^{HFD}+HFD or FMT^{HFD2}+HFD mice and in **(H)** HFD+FMT^{HFD} or HFD+FMT^{HFD2} mice. Data represent the mean±SEM of 9 or 12 mice.

Table S1. List of oligonucleotides used in qPCR.

Target	5' Forward 3'	5' Reverse 3'
18s	GTA-ACC-CGT-TGA-ACC-CCA-TT	CCA-TCC-AAT-CGG-TAG-TAG-CG
Ccl2	AGG-TCC-CTG-TCA-TGC-TTC-TG	TCT-GGA-CCC-ATT-CCT-TCT-TG
Cidea	GCA-GCC-TGC-AGG-AAC-TTA-TC	TCA-TGA-AAT-GCG-TGT-TGT-CC
CPT1	TCT-TGC-AGT-CGA-CTC-ACC-TT	TCC-ACA-GGA-CAC-ATA-GTC-AGG
F4/80	CTT-TGG-CTA-TGG-GCT-TCC-AGT-C	GCA-AGG-AGG-ACA-GAG-TTT-ATC-GTC
Gapdh	GTG-GAC-CTC-ATG-GCC-TAC-AT	TGT-GAG-GGA-GAT-GCT-CAG-TG
GPR41	CTG-GCG-GAG-CTA-CGT-GCT	GGG-GTC-GAT-ACA-AGA-GT
GPR43	CAC-GGC-CTA-CAT-CCT-CAT-CT	TTG-GTA-GGT-ACC-AGC-GGA-AG
UCP1	GCT-ACA-CGG-GGA-CCT-ACA-ATG	CGT-CAT-CTG-CCA-GTA-TTT-TGT-T

Table S2. GC/MS and quantitative parameters of SCFAs analysis. Linearity, calibration equation, limit of detection (LOD) and limit of quantification (LOQ).

SCFA species	*Mass (m/z)*	*Retention time (min)*	*Internal standards*	*Calibration equation (y=Ax+B)*	*R2*	*Linearity range (µM)*	*LOD (µM)**	*LOQ (µM)**
Acetate	43, 60	4.5	Acetate-D3	y = 0.008x + 0.003	0.993	2.3-500	2.3	2.39
Propionate	57, 75	5.9	Propionate-D2	y = 0.013x + 0.267	0.997	1.2-200	1.09	1.23
Isobutyrate	43, 71	6.9	Butyrate-13C2	y = 0.017x + 0.021	0.994	0.3-100	0.23	0.35
Butyrate	43, 71	7.7	Butyrate-13C2	y = 0.003x + 0.013	0.973	1.3-200	0.69	1.3
Isovalerate	73, 85	8.6	Valerate-D9	y = 0.035x + 0.001	0.998	0.2-100	0.15	0.26
Valerate	73, 85	9.2	Valerate-D9	y = 0.036x + 0.009	0.998	0.2-100	0.11	0.22

**LOD=Mb + 3xSDb ;LOQ=Mblank+ 10xSD blank; where Mb is the mean concentration of the blank and SDb is the standard deviation of the blank*
